# Considering prediagnostic environmental modifiers of progression in amyotrophic lateral sclerosis

**DOI:** 10.1136/bmjno-2026-001702

**Published:** 2026-05-29

**Authors:** Christos V Chalitsios, Martin R Turner

**Affiliations:** 1 Nuffield Department of Clinical Neurosciences, University of Oxford, Oxford, UK

**Keywords:** EPIDEMIOLOGY, MOTOR NEURON DISEASE, ALS

 In 1956, Manchester-based neurologist Liversedge introduced the neurodegenerative disorder amyotrophic lateral sclerosis (ALS), the most common form of motor neuron disease, noting: “The origin of the disorder has been attributed to a curious medley of factors, including chill, anxiety, toxicosis, injury, and inevitably tobacco and alcohol.”^1^ 70 years of intense epidemiological study since then has not answered this origin question definitively despite a large range of candidate factors. Observations of geographically isolated clusters of complex neurodegenerative disorders with an ALS phenotype in Guam and the Kii Peninsula focused on possible dietary neurotoxins.^2 3^ There has been a recurring focus on an association of athleticism in those developing ALS (see commentary Turner^4^), with the study of other factors, including but not limited to smoking and alcohol consumption,^5^ head injury,^6^ occupational exposures,^7^ military service,^8^ heavy metals^9^ and air pollution.^10^ Advances in the understanding of the genetic architecture of ALS^11^ have led to the formulation that ALS reflects interactions between external exposures and an underlying biological susceptibility.^12^

Most of the epidemiological studies to date have focused on disease incidence. ALS is a notoriously clinically heterogeneous syndrome. Although median survival is 30 months from symptom onset, at least 5% of individuals survive more than 10 years.^13^ Whether environmental exposures might influence the trajectory of ALS once established has received far less attention. This distinction is critical. Determinants of disease onset are not necessarily determinants of disease progression, and conflation of the two risks obscures both biological insight and clinical utility. In this context, the study by Martinez-Nunez *et al*^14^ considered longitudinal data from more than 8000 individuals enrolled in the US National ALS Registry. The authors examined whether prediagnostic environmental and occupational exposures were associated with the rate of functional decline, measured using the current standard of the revised ALS Functional Rating Scale (ALSFRS-R), following diagnosis. The authors explicitly attempt to bridge the gap between environmental epidemiology and disease course, asking whether there is evidence that prior exposures leave a lasting imprint on the tempo of symptomatic neurodegeneration.

## What the study reports

The analysis leverages repeated ALSFRS-R measurements collected at 3-month intervals, modelled using mixed-effects regression with non-linear time terms. A cubic trajectory provided the best fit, capturing the well-recognised non-linearity of measures of ALS disability progression.^15^

Within this framework, three exposures—herbicides, metal dust/fumes and oil-based paints—were associated with a steeper decline over time. The magnitude of these effects, while modest, is not negligible. Herbicide exposure, for example, was associated with an additional decline of approximately 0.57 ALSFRS-R points per year. Against a baseline decline of 2.8 points annually, this represents a potentially meaningful acceleration. In contrast, prior head injury was associated with lower ALSFRS-R scores overall but did not modify the slope of decline. This pattern is compatible with the hypothesis that traumatic exposures might act earlier in the disease process, perhaps influencing susceptibility, timing of onset or prediagnostic progression, rather than altering the rate of decline once the disease is clinically manifest. The authors interpret these findings as evidence for a cumulative exposure burden influencing disease trajectory, with biologically plausible mechanisms centred on well-established candidate cellular pathways in ALS pathogenesis, including oxidative stress, mitochondrial dysfunction and TAR DNA-binding protein 43 (TDP-43) proteinopathy (the pathological hallmark of nearly all cases of ALS and the related disorder frontotemporal dementia^16^).

## Specific limitations

The sample size is substantial, the use of longitudinal data is appropriate and the modelling approach is more sophisticated than the simple slope-based analyses that have characterised much of the prior literature. The consistency of findings across alternative model specifications and sensitivity analyses adds a degree of robustness. However, several limitations substantially impact the strength of the conclusions that may be drawn.

### Exposure assessment

One important limitation is the reliance on self-reported exposure histories. Participants were classified as exposed if they recalled having worked with a given substance for at least 100 days at any point prior to enrolment. There is no information on intensity, cumulative dose, timing relative to disease onset or coexposures. This introduces multiple sources of bias. Recall bias is always a concern, particularly in a population with progressive neurological impairment. More subtly, exposure misclassification is likely to be non-differential but substantial, diluting true associations while also increasing the risk of spurious findings in the presence of multiple comparisons. The absence of objective exposure metrics, whether environmental monitoring data, job-exposure matrices or biomarker-based measures, remains a major challenge in this field and is not overcome here.

### Confounding

The models adjust for age, sex and time since diagnosis, but omit several key determinants of ALS progression, including genetic status, site of onset, respiratory function and treatment use. Each of these factors is associated with disease trajectory and may also correlate with occupational or environmental exposures. Socioeconomic status and healthcare access are particularly relevant. Individuals with occupational exposures to metal dust or paints may differ systematically in access to specialist care, nutritional support or disease-modifying therapies. Without adequate adjustment, the observed associations may reflect these underlying differences rather than a direct effect of exposure. The authors did not include education level on the grounds of potential collinearity. Excluding such variables may simplify model estimation but risks leaving important confounding unaddressed.

### Timing and disease stage

A further limitation relates to the timing of observation. Participants entered the study a median of 2 years after diagnosis and with a mean ALSFRS-R Score of approximately 26, indicating substantial functional impairment at baseline. As a result, the analysis primarily reflects later-stage disease progression. If environmental exposures exert their effects earlier in the disease course, as suggested by the head injury findings, this design may underestimate or mischaracterise their true impact. The use of time since diagnosis, rather than symptom onset, introduces additional uncertainty. Diagnostic delay varies across patients and may itself be influenced by disease aggressiveness, potentially biasing estimates of progression.

### Attrition and extrapolation

Longitudinal attrition is substantial, with relatively few participants contributing data beyond 3 years. While the use of mixed-effects models mitigates some of the resulting bias, the estimation of long-term trajectories, particularly within a cubic framework, relies increasingly on extrapolation. The observation that herbicide effects emerge only with longer follow-up is intriguing but should be interpreted cautiously in light of this limitation.

## Future study considerations

The study highlights both the potential and the methodological challenges of investigating environmental modifiers of ALS progression. Future work should prioritise objective measures, including biomarkers, environmental linkage data and validated job-exposure matrices. The integration of genetic data is essential. ALS is increasingly understood as a disease of gene–environment interaction and incorporating genomic information would allow identification of susceptible subgroups and help explain heterogeneity in progression. Finally, recruitment earlier in the disease course and deeper phenotyping are needed. Studying patients closer to symptom onset, with detailed longitudinal clinical data, would allow clearer separation of factors influencing disease initiation versus progression.

## Conclusion

Despite its limitations, this study offers an important shift in perspective on how environmental exposures may influence, not only whether ALS develops, but also how it then unfolds. The modest effect sizes and uncertainty regarding causality currently preclude the use of exposure history as a reliable prognostic tool. However, such findings, if validated, may have relevance for clinical trial design. If environmental exposures contribute to heterogeneity in progression, accounting for them in stratification or adjustment strategies could improve statistical power. The enduring appeal of environmental explanations for ALS reflects both scientific curiosity and a desire to identify modifiable factors in a devastating disease with no strong therapeutic options despite many trials. For now, the study serves as a reminder that the story of ALS is unlikely to be written solely in the genome as we currently understand it, nor solely in the environment, but in their complex interplay.
